# ﻿Diversity survey of *Pholcus* spiders (Araneae, Pholcidae) from eastern Sichuan and neighboring areas, with descriptions of six new species

**DOI:** 10.3897/zookeys.1240.147905

**Published:** 2025-06-03

**Authors:** Jinglin Li, Shuqiang Li, Xiaoqing Zhang, Zhiyuan Yao

**Affiliations:** 1 College of Life Science, Shenyang Normal University, Shenyang 110034, China Shenyang Normal University Shenyang China; 2 Institute of Zoology, Chinese Academy of Sciences, Beijing 100101, China Chinese Academy of Sciences Beijing China

**Keywords:** Biodiversity, cellar spider, DNA barcode, invertebrate, taxonomy

## Abstract

Thirteen spider species of the genus *Pholcus* Walckenaer, 1805 are reported from a diversity survey in eastern Sichuan and neighboring areas (northeastern Yunnan and western Guizhou). They belong to three species groups and include six newly described species: *Pholcusqiaojia* Li, Li & Yao, **sp. nov.** (♂♀, Yunnan) in the *bidentatus* group; *P.aba* Li, Li & Yao, **sp. nov.** (♂♀, Sichuan) and *P.wenchuan* Li, Li & Yao, **sp. nov.** (♂♀, Sichuan) in the *crypticolens* group; *P.mengding* Li, Li & Yao, **sp. nov.** (♂♀, Sichuan), *P.miyi* Li, Li & Yao, **sp. nov.** (♂♀, Sichuan) and *P.yaan* Li, Li & Yao, **sp. nov.** (♂♀, Sichuan) in the *yichengicus* group. *P.bidentatus* Zhu, Zhang, Zhang & Chen, 2005 is recorded from Yunnan for the first time and *P.kunming* Zhang & Zhu, 2009 is recorded from Guizhou and Sichuan for the first time. Detailed diagnoses, descriptions, photomicroscope images, and DNA barcodes of all newly described species are provided.

## ﻿Introduction

The family Pholcidae C.L. Koch, 1850 is a highly diverse group of spiders, with 97 genera and 2,024 species (WSC 2025). *Pholcus* Walckenaer, 1805 is the most diverse genus of the family and mainly distributed in the Afrotropical, Palaearctic, Indo-Malayan, and Australasian regions (e.g., Huber 2011; Yao and Li 2012, 2025; WSC 2025). This genus comprises 21 species groups and 409 species (Huber 2011; Huber et al. 2018; WSC 2025). China exhibits the highest species diversity of *Pholcus*. To date, 179 species have been recorded in China, which represent 44% of the genus (WSC 2025). Recently, a series of surveys of *Pholcus* have been carried out in China, based on morphological and molecular data. For instance, the extensive 2020 expedition into the Changbai Mountains brings the species count of *Pholcus* in the Changbai Mountains to 27 species, including 13 newly described species (Lu et al. 2021; Yao et al. 2021; Zhao et al. 2023a). The systematic investigation in the Yanshan-Taihang Mountains in 2021 recorded 36 *Pholcus* species, of which 14 species were new to science (Lu et al. 2022a, b). During an expedition in 2022 to the Lüliang Mountains, one known *Pholcus* species and eight newly described species were reported (Zhao et al. 2023b). In 2022, a survey of the Qinling Mountains reported 20 *Pholcus* species, including nine newly described species (Yang et al. 2024a, b). Nevertheless, these efforts have primarily focused on northern and central China, with few reports in southern China.

**Figure 1.**
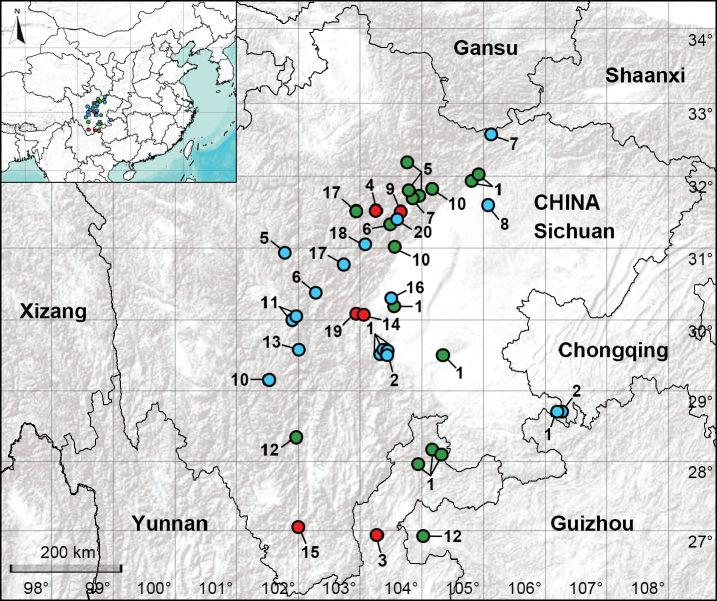
Distribution records of *Pholcus* spiders from the eastern of Sichuan and neighboring areas in this study. The *bidentatus* group: **1***P.bidentatus***2***P.kimi***3***P.qiaojia* sp. nov.; the *crypticolens* group: **4***P.aba* sp. nov. **5***P.chang***6***P.ganziensis***7***P.manueli***8***P.spilis***9***P.wenchuan* sp. nov.; the *yichengicus* group: **10***P.jiulong***11***P.kangding***12***P.kunming***13***P.luding***14***P.mengding* sp. nov. **15***P.miyi* sp. nov. **16***P.qingchengensis***17***P.qinghaiensis***18***P.taibaiensis***19***P.yaan* sp. nov. **20***P.zhangae*. Blue, green, and red circles represent previously recorded species, species collected in this study, and new species, respectively.

Sichuan is located in the southwest of China. The eastern region of Sichuan, unlike the western part which borders the Tibetan Plateau, is dominated by the vast Sichuan Basin and its surrounding highlands. Previously, 13 species of *Pholcus* have been recorded in eastern Sichuan. In the present study, we report 13 species from eastern Sichuan, northeastern Yunnan, and western Guizhou, six of which are newly described species: five from eastern Sichuan and one from northeastern Yunnan (Fig. 1).

## ﻿Materials and methods

All specimens were collected by X. Zhang, Y. Wang, and Q. Meng. Specimens of each new species were collected from a single population, and no obvious morphological differences were observed in either the males or the females. Moreover, the color patterns on their bodies are consistent between the two sexes. Therefore, we treat the males and females collected from a single population as one species. Specimens were examined and measured with a Leica M205 C stereomicroscope. Left male palps were photographed. Epigynes were photographed before dissection. Vulvae were photographed after treating them in a 10% warm solution of potassium hydroxide (KOH) to dissolve soft tissues. Images were captured with a Canon EOS 750D wide zoom digital camera (24.2 megapixels) mounted on the stereomicroscope mentioned above and assembled using Helicon Focus v. 3.10.3 image stacking software (Khmelik et al. 2005). All measurements are given in millimeters (mm). Leg measurements are shown as: total length (femur, patella, tibia, metatarsus, tarsus). Leg segments were measured on their dorsal side. The distribution map was generated with ArcGIS v. 10.2 (ESRI Inc.). The specimens studied are deposited in the College of Life Science, Shenyang Normal University (SYNU) in Liaoning, China.

Terminology and taxonomic descriptions follow Huber (2011) and Yao et al. (2015, 2021). The following abbreviations are used in the descriptions: **ALE** = anterior lateral eye, **AME** = anterior median eye, **PME** = posterior median eye, **L/d** = length/diameter ratio; used in the illustrations: **a** = appendix, **aa** = anterior arch, **b** = bulb, **da** = distal apophysis, **e** = embolus, **fa** = frontal apophysis, **kn** = knob, **pa** = proximo-lateral apophysis, **pp** = pore plate, **pr** = procursus, **u** = uncus.

DNA barcode sequences of new species were obtained. A partial fragment of the mitochondrial cytochrome oxidase subunit I (COI) gene was targeted using the following primers: forward: LCO1490 (5’-GGTCAACAAATCATAAAGATATTGG-3’) and reverse: HCO2198 (5’-TAAACTTCAGGGTGACCAAAAAATCA-3’) (Folmer et al. 1994). Additional information on extraction, amplification and sequencing procedures is provided in Yao et al. (2016).

## ﻿Results

A total of 13 species from eastern Sichuan, northeastern Yunnan, and western Guizhou were identified, including six newly described species. Of the seven known species, *P.bidentatus* Zhu, Zhang, Zhang & Chen, 2005 is recorded from Yunnan for the first time and *P.kunming* Zhang & Zhu, 2009 is recorded from Guizhou and Sichuan for the first time. A list of known species is provided in Table 1 and descriptions of all the new species are provided below. Of the 13 previously recorded species from eastern Sichuan, we identified six from our collection. We did not collect seven previously recorded species of eastern Sichuan due to the selection of sampling sites: *P.kangding* Zhang & Zhu, 2009, *P.kimi* Song & Zhu, 1994, *P.luding* Tong & Li, 2010, *P.qingchengensis* Gao, Gao & Zhu, 2002, *P.spilis* Zhu & Gong, 1991, *P.taibaiensis* Wang & Zhu, 1992 and *P.zhangae* Zhang & Zhu, 2009. The distribution records of all the species above are provided in Fig. 1. One DNA barcode sequence was obtained from each new species and all sequences are deposited in GenBank. The voucher numbers, GenBank accession numbers and other related information are given in Table 2.

**Table 1.** Information of the seven known species collected and identified.

Species	Voucher code	Collection locality
***bidentatus* group**		
*P.bidentatus*	2 ♂ (SYNU-Ar00141F–42F) 1 ♀ (SYNU-Ar00143F)	**Sichuan**, Mianyang, Jiangyou, Zhonghua Town, Laojunshan Scenic Spot; 32.025833°N, 104.925833°E; 845 m; 14 May 2024
	1 ♂ (SYNU-Ar00144F) 2 ♀ (SYNU-Ar00145F–46F)	**Sichuan**, Mianyang, Jiangyou, Yongsheng Town, Xinbei Village; 31.944167°N, 104.809722°E; 639 m; 16 May 2024
	2 ♂ (SYNU-Ar00147F–48F) 1 ♀ (SYNU-Ar00149F)	**Sichuan**, Chengdu, Pujiang County, Kakadian, Jinma Village; 30.191920°N, 103.549838°E; 624 m; 26 May 2024
	1 ♂ (SYNU-Ar00150F) 1 ♀ (SYNU-Ar00151F)	**Sichuan**, Zigong, Cao County, Gaoshiti Forest Park; 29.504962°N, 104.345949°E; 622 m; 27 May 2024
	2 ♂ (SYNU-Ar00152F–53F) 2 ♀ (SYNU-Ar00154F–55F)	Yunnan*, Zhaotong, Yanjin County, Diaolouzi; 28.095434°N, 104.308418°E; 645 m; 27 May 2024
	1 ♂ (SYNU-Ar00156F) 2 ♀ (SYNU-Ar00157F–58F)	Yunnan*, Zhaotong, Yanjin County, Zhonghe Town, Aitian Village; 28.174544°N, 104.165321°E; 675 m; 28 May 2024
	2 ♂ (SYNU-Ar00159F–60F) 1 ♀ (SYNU-Ar00161F)	Yunnan*, Zhaotong, Daguan County, Xiaoguanxi Village; 27.965522°N, 103.936600°E; 537 m; 30 May 2024
***crypticolens* group**		
*P.chang*	2 ♂ (SYNU-Ar00162F–63F) 1 ♀ (SYNU-Ar00164F)	**Sichuan**, Aba, Mao County, Chuanwen Road; 31.805000°N, 103.783889°E; 1570 m; 17 May 2024
	1 ♂ (SYNU-Ar00165F) 1 ♀ (SYNU-Ar00166F)	**Sichuan**, Aba, Songpan County, G213 Road; 32.191944°N, 103.756667°E; 2464 m; 17 May 2024
	1 ♂ (SYNU-Ar00167F) 1 ♀ (SYNU-Ar00168F)	**Sichuan**, Aba, Mao County, Mati Village; 31.731111°N, 103.952500°E; 1435 m; 18 May 2024
*P.ganziensis*	2 ♂ (SYNU-Ar00169F–70F) 1 ♀ (SYNU-Ar00171F)	**Sichuan**, Aba, Wenchuan County, Dayu Grange; 31.340115°N, 103.487172°E; 1177 m; 20 May 2024
*P.manueli*	1 ♂ (SYNU-Ar00172F) 1 ♀ (SYNU-Ar00173F)	**Sichuan**, Aba, Mao County, Chuanwen Road; 31.695000°N, 103.848056°E; 1536 m; 19 May 2024
***yichengicus* group**		
*P.jiulong*	2 ♂ (SYNU-Ar00174F–75F) 2 ♀ (SYNU-Ar00176F–77F)	**Sichuan**, Mianyang, Beichuan County, Dunqing Road, Zaoerping; 31.826667°N, 104.163889°E; 881 m; 18 May 2024
	1 ♂ (SYNU-Ar00178F) 1 ♀ (SYNU-Ar00179F)	**Sichuan**, Dujiangyan, Houzhi Grange; 31.026629°N, 103.562174°E; 860 m; 20 May 2024
*P.kunming*	1 ♂ (SYNU-Ar00180F) 2 ♀ (SYNU-Ar00181F–82F)	Guizhou*, Bijie, Weining County, Zhaiga Village; 26.924964°N, 104.013934°E; 2292 m; 1 Jun. 2024
	2 ♂ (SYNU-Ar00183F–84F) 2 ♀ (SYNU-Ar00185F–86F)	Sichuan*, Liangshan, Mianning County, Lizhuang Town, Dishuiyan; 28.345710°N, 101.964906°E; 2618 m; 10 Jun. 2024
*P.qinghaiensis*	1 ♂ (SYNU-Ar00187F) 2 ♀ (SYNU-Ar00188F–89F)	**Sichuan**, Aba, Li County, Shiziping; 31.516888°N, 102.933251°E; 2350 m; 19 May 2024

* indicates new provincial records.

**Table 2.** Voucher specimen information.

New species	Voucher code	GenBank accession number	Sequence length	Collection locality
*P.aba* sp. nov.	W373 (SYNU-Ar00477)	PV494966	624 bp	**Sichuan**, Aba, Li County
*P.mengding* sp. nov.	W365 (SYNU-Ar00484)	PV494967	624 bp	**Sichuan**, Yaan, Yucheng District
*P.miyi* sp. nov.	W366 (SYNU-Ar00488)	PV494965	624 bp	**Sichuan**, Panzhihua, Miyi County
*P.qiaojia* sp. nov.	W361 (SYNU-Ar00472)	PV494963	624 bp	**Yunnan**, Zhaotong, Qiaojia County
*P.wenchuan* sp. nov.	W367 (SYNU-Ar00480)	PV494962	624 bp	**Sichuan**, Aba, Wenchuan County
*P.yaan* sp. nov.	W364 (SYNU-Ar00492)	PV494964	624 bp	**Sichuan**, Yaan, Lushan County

### ﻿Taxonomic accounts

**Family Pholcidae C.L. Koch, 1850**

**Subfamily Pholcinae C.L. Koch, 1850**

#### ﻿Genus Pholcus

Taxon classificationAnimaliaAraneaePholcidae

Walckenaer, 1805

B36BC58D-7A73-5847-ADA4-407F3E02B395

##### Type species.

*Araneaphalangioides* Fuesslin, 1775.

###### ﻿*Pholcusbidentatus* species group

This species group was recognized by Huber (2011). It includes 36 previously described species and is distributed in southern China, as well as mainland Southeast Asia (e.g., Huber 2011; Dong et al. 2016a, b; Dong et al. 2017; Zhu et al. 2018; Lan et al. 2021). Among them, 18 species have been recorded from southern China. One species is newly described below.

#### ﻿ Pholcus qiaojia

Taxon classificationAnimaliaAraneaePholcidae

Li, Li & Yao sp. nov.

AF49E1FC-8661-5A9D-9643-12E11B9376AE

https://zoobank.org/938BAF51-927F-4AA0-8E9D-65BF5BAE1B7B

Figs 2 , 3 , 14A

##### Type material.

***Holotype***: China • ♂; Yunnan, Zhaotong, Qiaojia County, Laodian Town, Dingjia Village; 26.938345°N, 103.276833°E; alt. 1713 m; 2 Jun. 2024; X. Zhang, Y. Wang & Q. Meng leg.; SYNU-Ar00471. ***Paratypes***: China • 1 ♂; same data as for the holotype; SYNU-Ar00472 • 1 ♀; same data as for the holotype; SYNU-Ar00473.

##### Etymology.

The specific name refers to the type locality; noun in apposition.

##### Diagnosis.

**Figure 2.**
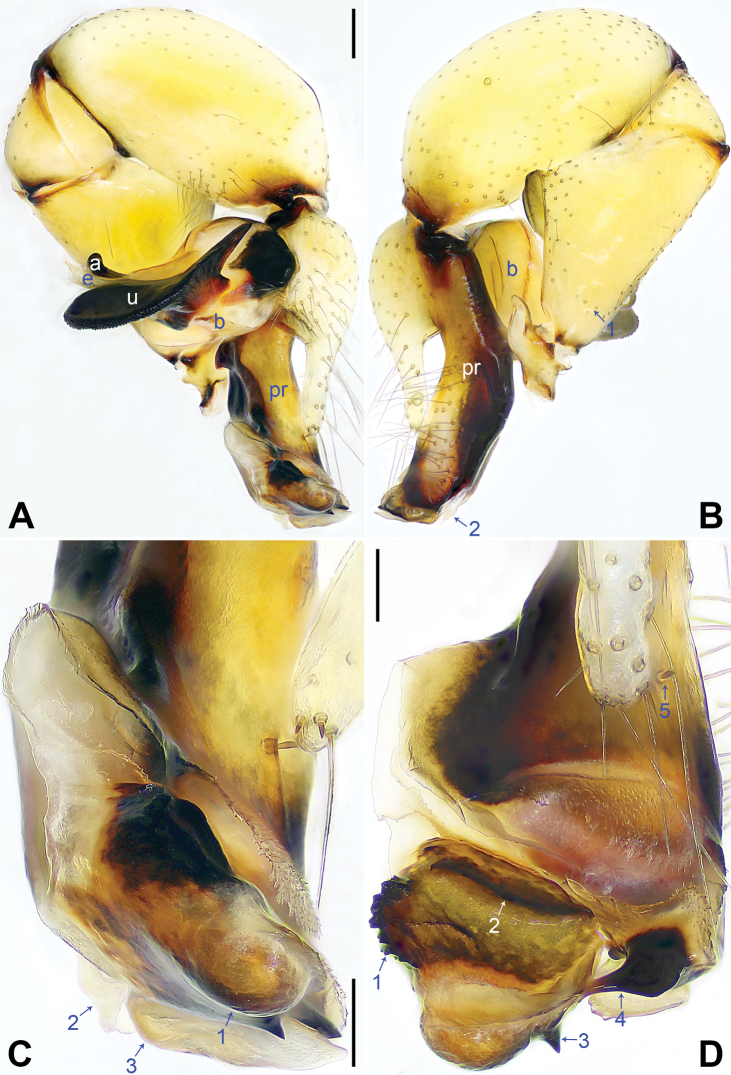
*Pholcusqiaojia* sp. nov., holotype male **A, B** palp (**A** prolateral view **B** retrolateral view, arrow 1 points at retrolatero-proximal protrusion, arrow 2 points at retrolatero-distal membranous process) **C, D** distal part of procursus (**C** prolateral view, arrow 1 points at blunt distal apophysis, arrow 2 points at retrolatero-distal membranous process, arrow 3 points at distal membranous process **D** dorsal view, arrow 1 points at sclerotized sawtooth apophyses, arrow 2 points at sclerotized flat apophysis, arrow 3 points at sclerotized pointed apophysis, arrow 4 points at sclerotized, proximally wide and distally pointed dorso-distal apophysis, arrow 5 points at dorsal spine). Abbreviations: a = appendix, b = bulb, e = embolus, pr = procursus, u = uncus. Scale bars: 0.20 (**A, B**); 0.10 (**C, D**).

**Figure 3.**
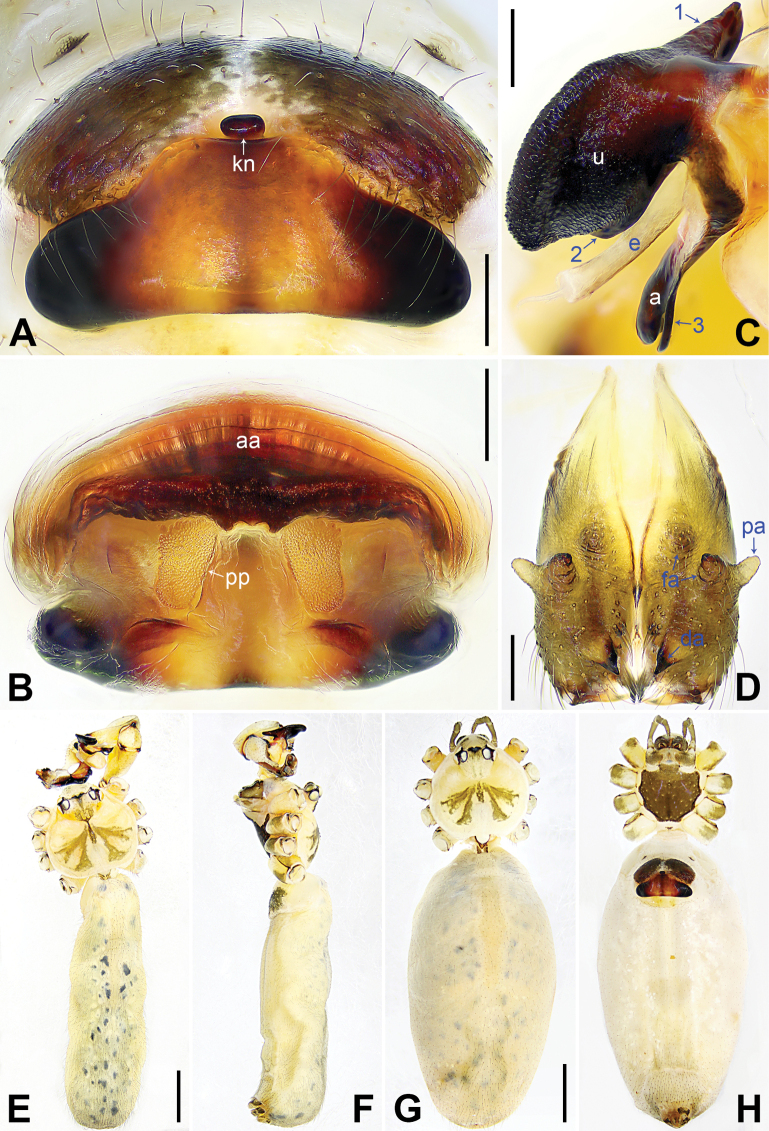
*Pholcusqiaojia* sp. nov., holotype (**D–F**) and paratype (**C**) males, paratype female (**A, B, G, H**) **A** epigyne, ventral view **B** vulva, dorsal view **C** bulbal apophyses, prolateral view, arrow 1 points at proximal apophysis, arrow 2 points at latero-median protrusion, arrow 3 points at slender subdistal branch **D** chelicerae, frontal view **E–H** habitus (**E, G** dorsal view **F** lateral view **H** ventral view). Abbreviations: a = appendix, aa = anterior arch, da = distal apophysis, e = embolus, fa = frontal apophysis, kn = knob, pa = proximo-lateral apophysis, pp = pore plate, u = uncus. Scale bars: 0.20 (**A–D**); 1.00 (**E–H**).

The new species resembles *P.bidentatus* Zhu, Zhang, Zhang & Chen, 2005 (Yao and Li 2012: 11, figs 33A–D, 34A–C; Huber 2011: 420, figs 1930, 1931, 1961, 1962, 2028–2033) by having similar uncus (Figs 3C, 14A) and epigyne (Fig. 3A), but can be distinguished by procursus with blunt distal apophysis (arrow 1 in Fig. 2C vs absent) and sclerotized sawtooth apophyses (arrow 1 in Fig. 2D vs absent), by male chelicerae with two pairs of frontal apophyses (fa in Fig. 3D vs absent), by appendix without angular median apophyses (a in Figs 3C, 14A vs with two), and by vulval pore plates 2× longer than wide (pp in Fig. 3B vs 3×).

##### Description.

**Male** (***holotype***): Measurements: Total length 6.88 (6.99 with clypeus), carapace 1.62 long, 1.84 wide, opisthosoma 5.26 long, 1.54 wide. Leg I: 51.81 (13.46, 0.78, 12.18, 22.76, 2.63), leg II: 33.82 (9.42, 0.75, 8.40, 13.65, 1.60), leg III: 23.79 (6.64, 0.70, 5.90, 9.30, 1.25), leg IV: missing; tibia I L/d: 81. Eye interdistances and diameters: PME–PME 0.40, PME 0.20, PME–ALE 0.06, AME–AME 0.09, AME 0.09. Sternum width/length: 1.16/0.94.

Color: Carapace yellowish, with brown radiating marks; ocular area yellowish, with median and lateral brown bands; clypeus yellowish, with indistinct brown marks; sternum brown. Legs yellowish, but dark brown on patellae and whitish on distal parts of femora and tibiae, with darker rings on subdistal parts of femora and proximal and subdistal parts of tibiae. Opisthosoma yellowish, with dorsal and lateral brown spots.

Body: Habitus as in Fig. 3E, F; ocular area elevated, each eye triad on top of laterally directed eye-stalk.

Chelicerae: As in Fig. 3D, with pair of proximo-lateral apophyses, pair of distal apophyses, and two pairs of frontal apophyses.

Palp: As in Fig. 2A, B; trochanter with long (3× longer than wide), retrolaterally bulged ventral apophysis; femur with retrolatero-proximal protrusion (arrow 1 in Fig. 2B) and distinct ventral protrusion; procursus simple proximally but complex distally, with raised, prolatero-subdistal membranous edge bearing blunt distal apophysis (arrow 1 in Fig. 2C) with sclerotized sawtooth apophyses, sclerotized flat apophysis and sclerotized pointed apophysis (arrows 1–3 in Fig. 2D), retrolatero-distal membranous process (arrow 2 in Fig. 2B, C), distal membranous process (arrow 3 in Fig. 2C), sclerotized, proximally wide and distally pointed dorso-distal apophysis (arrow 4 in Fig. 2D), and strong dorsal spine (arrow 5 in Fig. 2D); uncus with scales, proximal apophysis, and distinct latero-median protrusion (arrows 1, 2 in Figs 3C, 14A); appendix hooked, with slender subdistal branch (arrow 3 in Figs 3C, 14A); embolus weakly sclerotized, with some transparent distal projections (Figs 3C, 14A).

Legs: Retrolateral trichobothrium on tibia I at 2% proximally; legs with short vertical setae on tibiae, metatarsi, and tarsi; tarsus I with 42 distinct pseudosegments.

**Female** (***paratype***, SYNU-Ar00473): Similar to male, habitus as in Fig. 3G, H. Total length 6.46 (6.62 with clypeus), carapace 1.52 long, 1.70 wide, opisthosoma 4.94 long, 2.64 wide; leg I missing. Eye interdistances and diameters: PME–PME 0.25, PME 0.18, PME–ALE 0.04, AME–AME 0.04, AME 0.09. Sternum width/length: 1.10/1.03. Ocular area without eye-stalks. Epigyne nearly trapezoidal, laterally strongly sclerotized, with knob (Fig. 3A). Vulva with curved, posteriorly sclerotized anterior arch and pair of nearly quadrilateral pore plates (Fig. 3B).

##### Variation.

Unknown. Leg I missing in paratype male (SYNU-Ar00472).

##### Habitat.

Underside of overhang on rocky cliffs in the mountain area.

##### Distribution.

China (Yunnan, type locality; Fig. 1).

###### ﻿*Pholcuscrypticolens* species group

This species group was recognized by Huber (2011). It includes 11 previously described species and is mainly distributed in East Asia (e.g., Huber 2011; Yao and Li 2012; Dong et al. 2016b). Among them, 10 species have been recorded from China. Two species are newly described below.

#### ﻿ Pholcus aba

Taxon classificationAnimaliaAraneaePholcidae

Li, Li & Yao sp. nov.

89DE8712-9E22-5300-8F5B-1729EC6F832C

https://zoobank.org/45EE1D92-D2A2-4F24-AF74-7AA6CD0B35BF

Figs 4 , 5 , 14B

##### Type material.

***Holotype***: China • ♂; Sichuan, Aba, Li County, Xindianzi; 31.528726°N, 103.254276°E; alt. 1606 m; 19 May 2024; X. Zhang, Y. Wang & Q. Meng leg.; SYNU-Ar00474. ***Paratypes***: China • 1 ♂; same data as for the holotype; SYNU-Ar00475 • 2 ♀; same data as for the holotype; SYNU-Ar00476–77.

##### Etymology.

The specific name refers to the type locality; noun in apposition.

##### Diagnosis.

**Figure 4.**
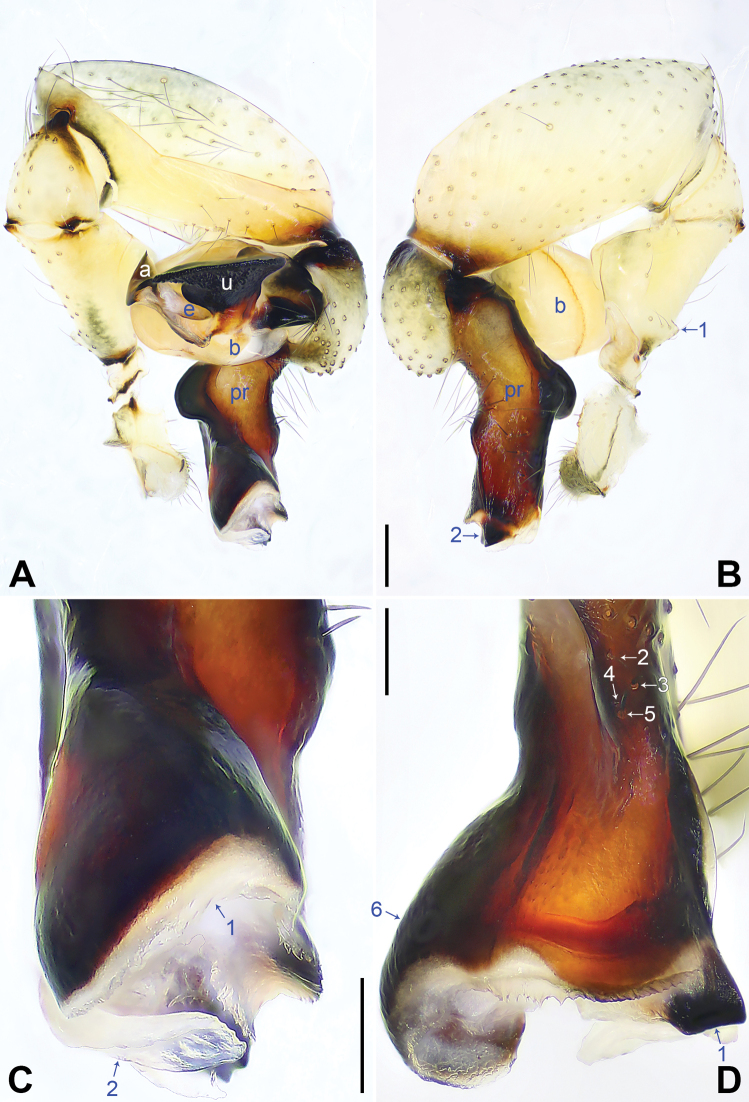
*Pholcusaba* sp. nov., holotype male **A, B** palp (**A** prolateral view **B** retrolateral view, arrow 1 points at retrolatero-proximal protrusion, arrow 2 points at sclerotized retrolatero-distal apophysis) **C, D** distal part of procursus (**C** prolateral view, arrow 1 points at membranous part, arrow 2 points at distal membranous process **D** dorsal view, arrow 1 points at sclerotized retrolatero-distal apophysis, arrows 2–5 point at dorsal spines, arrow 6 points at swollen prolatero-subdistal edge). Abbreviations: a = appendix, b = bulb, e = embolus, pr = procursus, u = uncus. Scale bars: 0.20 (**A, B**); 0.10 (**C, D**).

**Figure 5.**
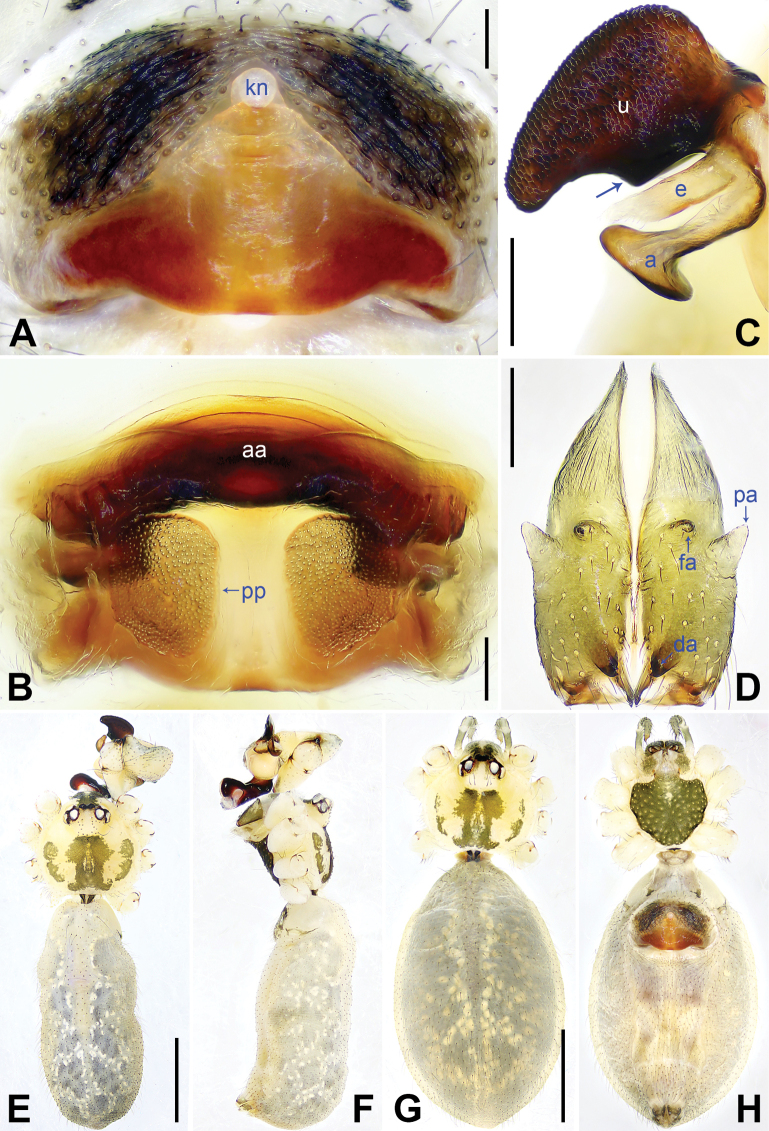
*Pholcusaba* sp. nov., holotype male (**C–F**) and paratype female (**A, B, G, H**) **A** epigyne, ventral view **B** vulva, dorsal view **C** bulbal apophyses, prolateral view, arrow points at latero-median protrusion **D** chelicerae, frontal view **E–H** habitus (**E, G** dorsal view **F** lateral view **H** ventral view). Abbreviations: a = appendix, aa = anterior arch, da = distal apophysis, e = embolus, fa = frontal apophysis, kn = knob, pa = proximo-lateral apophysis, pp = pore plate, u = uncus. Scale bars: 0.10 (**A, B**); 0.20 (**C, D**); 1.00 (**E–H**).

The new species resembles *P.langensis* Yao & Li, 2016 (Dong et al. 2016b: 8, figs 5A–D, 6A–H) by having similar uncus (Figs 5C, 14B), male chelicerae (Fig. 5D) and epigyne (Fig. 5A), but can be distinguished by procursus with raised, sclerotized, swollen prolatero-subdistal edge (arrow 6 in Fig. 4D vs prolatero-subdistal edge not swollen), sclerotized retrolatero-distal apophysis (arrow 2 in Fig. 4B; arrow 1 in Fig. 4D vs absent) and distal membranous process without sawteeth (arrow 2 in Fig. 4C vs present), by appendix without median apophyses (a in Figs 5C, 14B vs with sawtooth median apophyses), and by vulval pore plates longer than wide (pp in Fig. 5B vs wider than long).

##### Description.

**Male** (***holotype***): Measurements: Total length 3.90 (4.03 with clypeus), carapace 1.12 long, 1.25 wide, opisthosoma 2.78 long, 1.24 wide. Leg I: 24.32 (6.28, 0.52, 6.41, 9.23, 1.88), leg II: 16.59 (4.70, 0.51, 4.25, 6.03, 1.10), leg III: 12.47 (3.72, 0.49, 3.09, 4.35, 0.82), leg IV: 15.81 (4.80, 0.50, 4.16, 5.32, 1.03); tibia I L/d: 51. Eye interdistances and diameters: PME–PME 0.20, PME 0.13, PME–ALE 0.03, AME–AME 0.04, AME 0.07. Sternum width/length: 0.89/0.74.

Color: Carapace yellowish, with median brown marks and marginal brown bands; ocular area yellowish; clypeus yellowish, with brown marks; sternum brown. Legs yellowish, but brownish on patellae and whitish on distal parts of femora and tibiae, with darker rings on subdistal parts of femora and proximal and subdistal parts of tibiae. Opisthosoma yellowish, with light dorsal and lateral spots.

Body: Habitus as in Fig. 5E, F; ocular area elevated, without eye-stalks.

Chelicerae: As in Fig. 5D, with pair of proximo-lateral apophyses, pair of distal apophyses with two teeth each, and pair of frontal apophyses.

Palp: As in Fig. 4A, B; trochanter with long (3× longer than wide), retrolaterally strongly bulged ventral apophysis; femur with retrolatero-proximal protrusion (arrow 1 in Fig. 4B) and distinct ventral protrusion; procursus simple proximally but complex distally, with raised, sclerotized, swollen prolatero-subdistal edge bearing membranous part (arrow 6 in Fig. 4D; arrow 1 in Fig. 4C), distal membranous process (arrow 2 in Fig. 4C), sclerotized retrolatero-distal apophysis (arrow 1 in Fig. 4D; arrow 2 in Fig. 4B), and one slender and three strong dorsal spines (arrows 2–5 in Fig. 4D); uncus with scales, proximal apophysis, and distinct latero-median protrusion (arrow in Fig. 5C; arrows 1, 2 in Fig. 14B); appendix T-shaped (Figs 5C, 14B); embolus weakly sclerotized, with some transparent distal projections (Figs 5C, 14B).

Legs: Retrolateral trichobothrium on tibia I at 6% proximally; legs with short vertical setae on tibiae, metatarsi, and tarsi; tarsus I with 27 distinct pseudosegments.

**Female** (***paratype***, SYNU-Ar00476): Similar to male, habitus as in Fig. 5G, H. Total length 4.05 (4.18 with clypeus), carapace 1.08 long, 1.25 wide, opisthosoma 2.97 long, 1.82 wide; tibia I: 5.96; tibia I L/d: 46. Eye interdistances and diameters: PME–PME 0.17, PME 0.13, PME–ALE 0.03, AME–AME 0.04, AME 0.06. Sternum width/length: 0.82/0.76. Epigyne nearly triangular, laterally strongly sclerotized, with knob (Fig. 5A). Vulva with curved, posteriorly sclerotized anterior arch and pair of nearly round pore plates (Fig. 5B).

##### Variation.

Tibia I in paratype male (SYNU-Ar00475): 6.92. Tibia I in another paratype female (SYNU-Ar00477): 6.41.

##### Habitat.

Underside of overhang on rocky cliffs in the mountain area.

##### Distribution.

China (Sichuan, type locality; Fig. 1).

#### ﻿ Pholcus wenchuan

Taxon classificationAnimaliaAraneaePholcidae

Li, Li & Yao sp. nov.

FB1631B9-8401-552C-90C0-06F8382BAB55

https://zoobank.org/EE68B9CF-B08C-4661-9480-4A84C5ED8062

Figs 6 , 7 , 14C

##### Type material.

***Holotype***: China • ♂; Sichuan, Aba, Wenchuan County, Qingpo Village; 31.512222°N, 103.661667°E; alt. 1389 m; 19 May 2024; X. Zhang, Y. Wang & Q. Meng leg.; SYNU-Ar00478. ***Paratypes***: China • 1 ♂; same data as for the holotype; SYNU-Ar00479 • 1 ♀; same data as for the holotype; SYNU-Ar00480.

##### Etymology.

The specific name refers to the type locality; noun in apposition.

##### Diagnosis.

**Figure 6.**
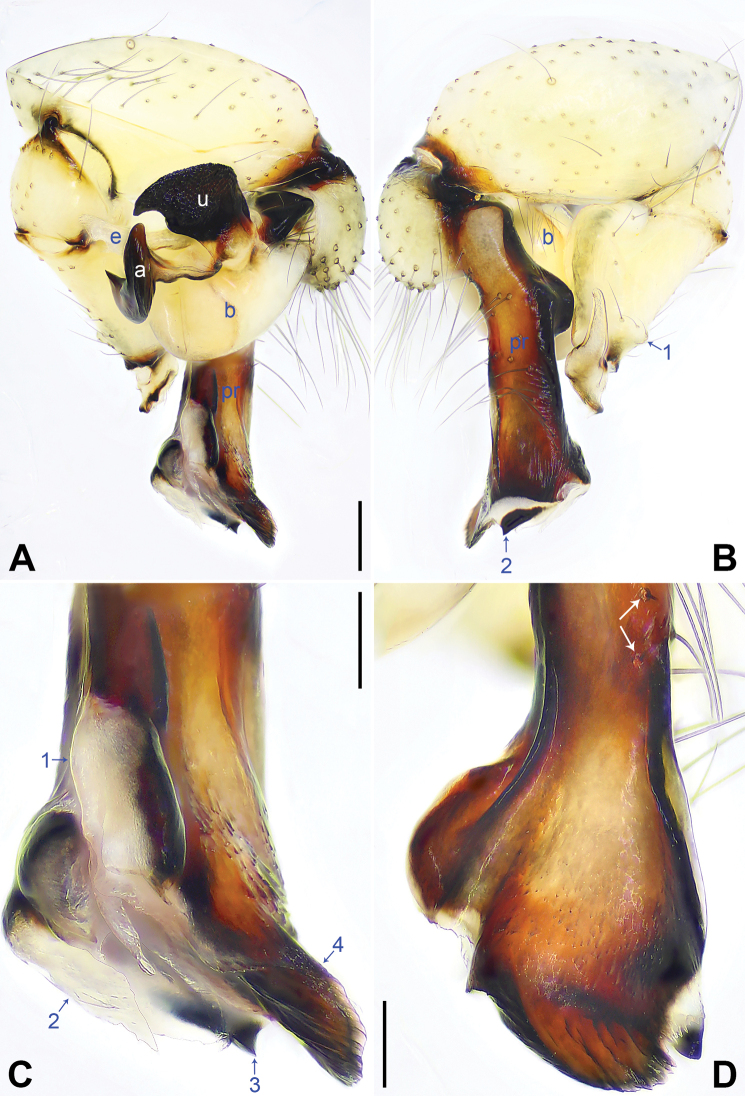
*Pholcuswenchuan* sp. nov., holotype male **A, B** palp (**A** prolateral view **B** retrolateral view, arrow 1 points at retrolatero-proximal protrusion, arrow 2 points at sclerotized retrolatero-distal apophysis) **C, D** distal part of procursus (**C** prolateral view, arrow 1 points at membranous part, arrow 2 points at distal membranous process, arrow 3 points at sclerotized retrolatero-distal apophysis, arrow 4 points at curved dorso-distal sclerite **D** dorsal view, arrows point at dorsal spines). Abbreviations: a = appendix, b = bulb, e = embolus, pr = procursus, u = uncus. Scale bars: 0.20 (**A, B**); 0.10 (**C, D**).

**Figure 7.**
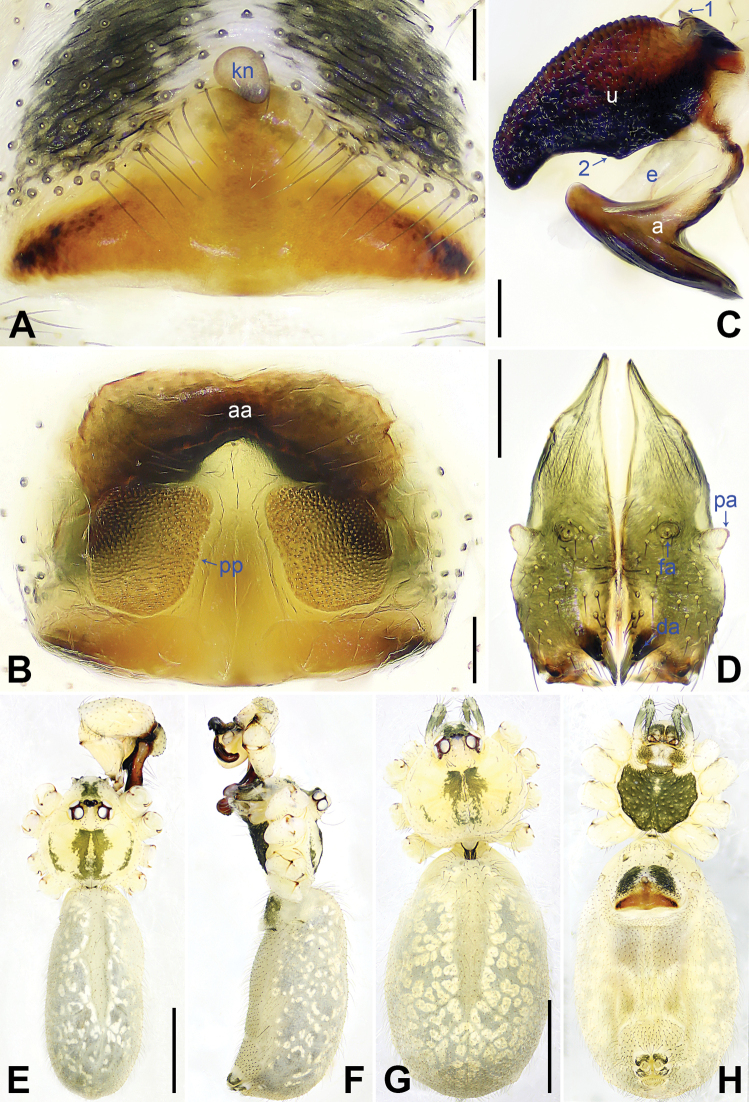
*Pholcuswenchuan* sp. nov., holotype male (**C–F**) and paratype female (**A, B, G, H**) **A** epigyne, ventral view **B** vulva, dorsal view **C** bulbal apophyses, prolateral view, arrow 1 points at proximal apophysis, arrow 2 points at latero-median protrusion **D** chelicerae, frontal view **E–H** habitus (**E, G** dorsal view **F** lateral view **H** ventral view). Abbreviations: a = appendix, aa = anterior arch, da = distal apophysis, e = embolus, fa = frontal apophysis, kn = knob, pa = proximo-lateral apophysis, pp = pore plate, u = uncus. Scale bars: 0.10 (**A–C**); 0.20 (**D**); 1.00 (**E–H**).

The new species resembles *P.ganziensis* Yao & Li, 2012 (Yao and Li 2012: 16, figs 65A–D, 66A–E, 67A–D, 68A–D) by having similar bulbal apophyses (Figs 7C, 14C), male chelicerae (Fig. 7D) and epigyne (Fig. 7A), but can be distinguished by procursus with curved and elongated dorso-distal sclerite (arrow 4 in Fig. 6C vs flat, nearly angular, and not elongated) and raised, sclerotized prolatero-subdistal edge bearing membranous part (arrow 1 in Fig. 6C vs prolatero-subdistal edge without membranous part) and by vulval pore plates nearly quadrilateral (pp in Fig. 7B vs nearly round).

##### Description.

**Male** (***holotype***): Measurements: Total length 3.66 (3.80 with clypeus), carapace 1.16 long, 1.25 wide, opisthosoma 2.50 long, 1.15 wide. Leg I: 25.27 (6.60, 0.52, 6.54, 9.81, 1.80), leg II: 17.19 (4.80, 0.49, 4.40, 6.35, 1.15), leg III: 12.76 (3.80, 0.48, 3.09, 4.50, 0.89), leg IV: 16.90 (5.00, 0.51, 4.40, 5.96, 1.03); tibia I L/d: 50. Eye interdistances and diameters: PME–PME 0.16, PME 0.14, PME–ALE 0.03, AME–AME 0.04, AME 0.06. Sternum width/length: 0.88/0.79.

Color: Carapace yellowish, with median brown marks and marginal brown bands; ocular area yellowish; clypeus yellowish, with brown marks; sternum brown. Legs yellowish, but brownish on patellae and whitish on distal parts of femora and tibiae, with darker rings on subdistal parts of femora and proximal and subdistal parts of tibiae. Opisthosoma yellowish, with light dorsal and lateral spots.

Body: Habitus as in Fig. 7E, F; ocular area elevated, without eye-stalks.

Chelicerae: As in Fig. 7D, with pair of proximo-lateral apophyses, pair of distal apophyses with two teeth each, and pair of frontal apophyses.

Palp: As in Fig. 6A, B; trochanter with long (4× longer than wide), retrolaterally strongly bulged ventral apophysis; femur with retrolatero-proximal protrusion (arrow 1 in Fig. 6B) and distinct ventral protrusion; procursus simple proximally but complex distally, with raised, sclerotized prolatero-subdistal edge bearing membranous part (arrow 1 in Fig. 6C), distal membranous process (arrow 2 in Fig. 6C), sclerotized retrolatero-distal apophysis (arrow 3 in Fig. 6C; arrow 2 in Fig. 6B), curved dorso-distal sclerite (arrow 4 in Fig. 6C), and two strong dorsal spines (arrows in Fig. 6D); uncus with scales, proximal apophysis, and distinct latero-median protrusion (arrows 1, 2 in Figs 7C, 14C); appendix T-shaped (Figs 7C, 14C); embolus weakly sclerotized, with some transparent distal projections (Figs 7C, 14C).

Legs: Retrolateral trichobothrium on tibia I at 6% proximally; legs with short vertical setae on tibiae, metatarsi, and tarsi; tarsus I with 35 distinct pseudosegments.

**Female** (***paratype***, SYNU-Ar00480): Similar to male, habitus as in Fig. 7G, H. Total length 3.92 (4.06 with clypeus), carapace 1.20 long, 1.33 wide, opisthosoma 2.72 long, 1.90 wide; tibia I: 5.90; tibia I L/d: 49. Eye interdistances and diameters: PME–PME 0.16, PME 0.13, PME–ALE 0.03, AME–AME 0.03, AME 0.04. Sternum width/length: 0.84/0.70. Epigyne nearly triangular, laterally strongly sclerotized, with knob (Fig. 7A). Vulva with curved, sclerotized anterior arch and pair of nearly quadrilateral pore plates (Fig. 7B).

##### Variation.

Unknown. Leg I missing in paratype male (SYNU-Ar00479).

##### Habitat.

Underside of overhang on rocky cliffs in the mountain area.

##### Distribution.

China (Sichuan, type locality; Fig. 1).

###### ﻿*Pholcusyichengicus* species group

This species group was recognized by Huber (2011). It includes 48 previously described species and is mainly distributed in central and southern China, as well as northern Thailand (e.g., Huber 2011; Zhu et al. 2018; Lu et al. 2022b; Li et al. 2024; Yang et al. 2024a, b). Among them, 44 species have been recorded from China. Three species are newly described below.

#### ﻿ Pholcus mengding

Taxon classificationAnimaliaAraneaePholcidae

Li, Li & Yao sp. nov.

1855283F-EF52-5D46-B69F-A821C3C18456

https://zoobank.org/89E415FB-47EA-444E-9A95-32622D7AF360

Figs 8 , 9 , 14D

##### Type material.

***Holotype***: China • ♂; Sichuan, Yaan, Yucheng District, Mengdingshan Scenic Spot; 30.075869°N, 103.052207°E; alt. 982 m; 23 May 2024; X. Zhang, Y. Wang & Q. Meng leg.; SYNU-Ar00481. ***Paratypes***: China • 1 ♂; same data as for the holotype; SYNU-Ar00482 • 2 ♀; same data as for the holotype; SYNU-Ar00483–84.

##### Etymology.

The specific name refers to the type locality; noun in apposition.

##### Diagnosis.

**Figure 8.**
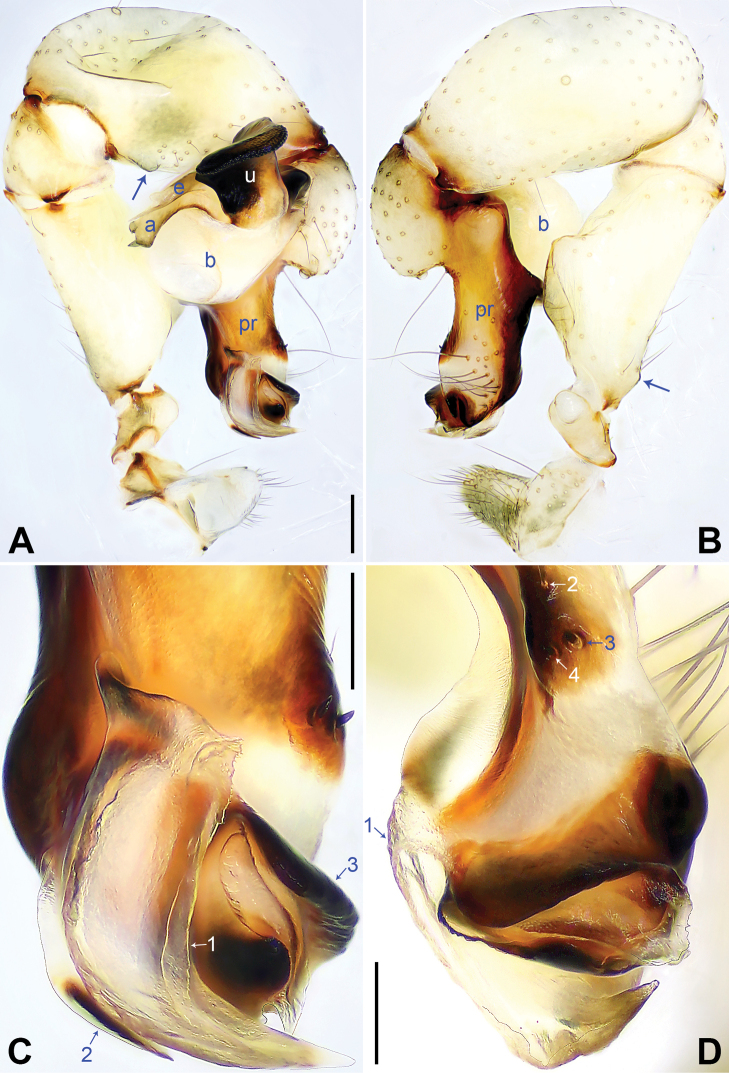
*Pholcusmengding* sp. nov., holotype male **A, B** palp (**A** prolateral view, arrow points at prolatero-ventral protrusion **B** retrolateral view, arrow points at retrolatero-proximal protrusion) **C, D** distal part of procursus (**C** prolateral view, arrow 1 points at distal membranous process, arrow 2 points at sclerotized part, arrow 3 points at sclerotized dorso-subdistal apophysis **D** dorsal view, arrow 1 points at laterally blunt prolatero-subdistal membranous edge, arrows 2–4 point at dorsal spines). Abbreviations: a = appendix, b = bulb, e = embolus, pr = procursus, u = uncus. Scale bars: 0.20 (**A, B**); 0.10 (**C, D**).

**Figure 9.**
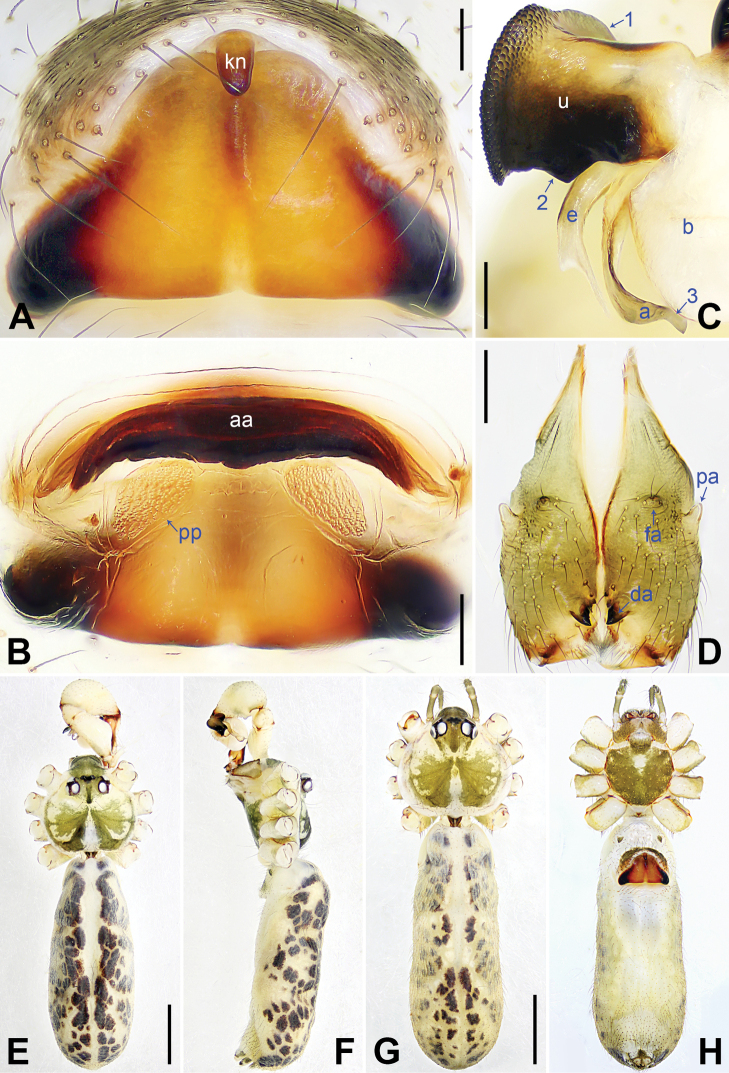
*Pholcusmengding* sp. nov., holotype male (**C–F**) and paratype female (**A, B, G, H**) **A** epigyne, ventral view **B** vulva, dorsal view **C** bulbal apophyses, prolateral view, arrow 1 points at proximal apophysis, arrow 2 points at latero-median protrusion, arrow 3 points at curved subdistal branch **D** chelicerae, frontal view **E–H** habitus (**E, G** dorsal view **F** lateral view **H** ventral view). Abbreviations: a = appendix, aa = anterior arch, b = bulb, da = distal apophysis, e = embolus, fa = frontal apophysis, kn = knob, pa = proximo-lateral apophysis, pp = pore plate, u = uncus. Scale bars: 0.10 (**A–C**); 0.20 (**D**); 1.00 (**E–H**).

The new species resembles *P.yaan* sp. nov. (Figs 12A–D, 13A–H, 14F) by having similar uncus (Figs 9C, 14D), male chelicerae (Fig. 9D) and epigyne (Fig. 9A), but can be distinguished by prolatero-subdistal membranous edge of procursus laterally blunt (arrow 1 in Fig. 8D vs laterally pointed), by appendix with curved subdistal branch (arrow 3 in Fig. 9C, arrow 4 in Fig. 14D vs absent), and by vulval pore plates nearly elliptic (2× longer than wide, pp in Fig. 9B vs nearly round).

##### Description.

**Male** (***holotype***): Measurements: Total length 5.08 (5.28 with clypeus), carapace 1.44 long, 1.66 wide, opisthosoma 3.64 long, 1.50 wide. Leg I: 49.94 (12.82, 0.71, 12.50, 21.35, 2.56), leg II: 32.27 (8.85, 0.67, 8.21, 12.82, 1.72), leg III: 22.07 (6.41, 0.64, 5.39, 8.33, 1.30), leg IV: 29.16 (8.33, 0.65, 7.24, 11.54, 1.40); tibia I L/d: 83. Eye interdistances and diameters: PME–PME 0.32, PME 0.17, PME–ALE 0.05, AME–AME 0.08, AME 0.08. Sternum width/length: 1.03/0.99.

Color: Carapace yellowish, with brown radiating marks and marginal brown bands; ocular area brownish, with median brown band; clypeus brown; sternum yellowish, with brown marks. Legs yellowish, but dark brown on patellae and whitish on distal parts of femora and tibiae, with darker rings on subdistal parts of femora and proximal and subdistal parts of tibiae. Opisthosoma yellowish, with dorsal and lateral brown spots.

Body: Habitus as in Fig. 9E, F; ocular area elevated, each eye triad on top of laterally directed eye-stalk.

Chelicerae: As in Fig. 9D, with pair of proximo-lateral apophyses, pair of distal apophyses with two teeth each, and pair of frontal apophyses.

Palp: As in Fig. 8A, B; trochanter with long (2× longer than wide), retrolaterally strongly bulged ventral apophysis; femur with retrolatero-proximal protrusion (arrow in Fig. 8B) and distinct ventral protrusion; tibia with prolatero-ventral protrusion (arrow in Fig. 8A); procursus simple proximally but complex distally, with raised, prolatero-subdistal membranous edge bearing distal membranous process (arrow 1 in Fig. 8C), distal membranous process bearing sclerotized part (arrow 2 in Fig. 8C), sclerotized dorso-subdistal apophysis (arrow 3 in Fig. 8C), and one slender and two strong dorsal spines (arrows 2–4 in Fig. 8D); uncus with scaly edge, proximal apophysis, and distinct latero-median protrusion (arrows 1, 2 in Figs 9C, 14D); appendix hooked, with curved subdistal branch (arrow 3 in Fig. 9C; arrows 3, 4 in Fig. 14D); embolus weakly sclerotized, with some transparent distal projections (Figs 9C, 14D).

Legs: Retrolateral trichobothrium on tibia I at 2% proximally; legs with short vertical setae on tibiae, metatarsi, and tarsi; tarsus I with 34 distinct pseudosegments.

**Female** (***paratype***, SYNU-Ar00483): Similar to male, habitus as in Fig. 9G, H. Total length 5.02 (5.22 with clypeus), carapace 1.42 long, 1.66 wide, opisthosoma 3.60 long, 1.34 wide; tibia I: 8.91; tibia I L/d: 64. Eye interdistances and diameters: PME–PME 0.22, PME 0.17, PME–ALE 0.05, AME–AME 0.06, AME 0.08. Sternum width/length: 1.01/0.93. Ocular area with median and lateral brown bands, without eye-stalks. Epigyne nearly trapezoidal, laterally strongly sclerotized, with knob (Fig. 9A). Vulva with curved, posteriorly sclerotized anterior arch and pair of nearly elliptic pore plates (Fig. 9B).

##### Variation.

Tibia I in paratype male (SYNU-Ar00482): 11.67. Tibia I in another paratype female (SYNU-Ar00484): 8.40.

##### Habitat.

Underside of overhang on rocky cliffs in the mountain area.

##### Distribution.

China (Sichuan, type locality; Fig. 1).

#### ﻿ Pholcus miyi

Taxon classificationAnimaliaAraneaePholcidae

Li, Li & Yao sp. nov.

B7177979-0B8B-5A51-A56C-4D66C5A8081A

https://zoobank.org/1CE5EE1D-9465-4006-8574-3F3BAB530911

Figs 10 , 11 , 14E

##### Type material.

***Holotype***: China • ♂; Sichuan, Panzhihua, Miyi County, Puwei Town, Pengjiayakou Village; 27.060020°N, 102.000282°E; alt. 2464 m; 5 Jun. 2024; X. Zhang, Y. Wang & Q. Meng leg.; SYNU-Ar00485. ***Paratypes***: China • 1 ♂; same data as for the holotype; SYNU-Ar00486 • 2 ♀; same data as for the holotype; SYNU-Ar00487–88.

##### Etymology.

The specific name refers to the type locality; noun in apposition.

##### Diagnosis.

**Figure 10.**
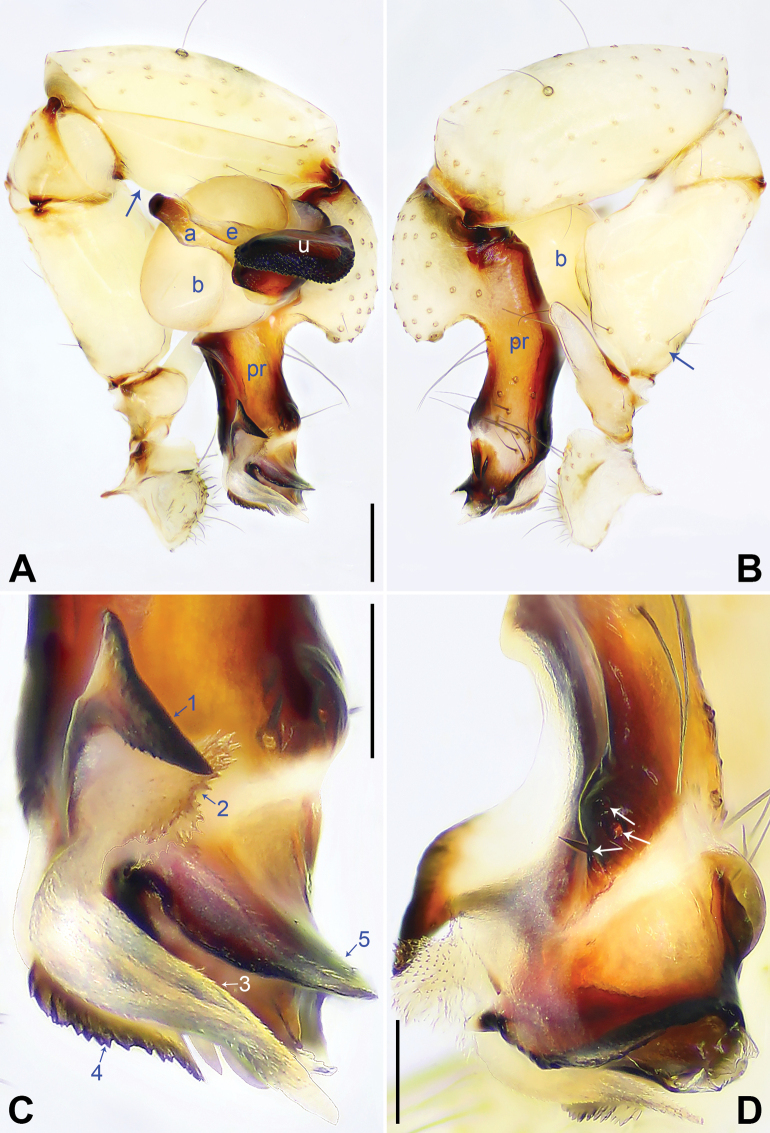
*Pholcusmiyi* sp. nov., holotype male **A, B** palp (**A** prolateral view, arrow points at prolatero-ventral protrusion **B** retrolateral view, arrow points at retrolatero-proximal protrusion) **C, D** distal part of procursus (**C** prolateral view, arrow 1 points at sclerotized pointed subdistal apophysis, arrow 2 points at sawtooth subdistal membranous process, arrow 3 points at distal membranous process, arrow 4 points at sawtooth distal apophysis, arrow 5 points at dorso-subdistal sclerite **D** dorsal view, arrows point at dorsal spines). Abbreviations: a = appendix, b = bulb, e = embolus, pr = procursus, u = uncus. Scale bars: 0.20 (**A, B**); 0.10 (**C, D**).

**Figure 11.**
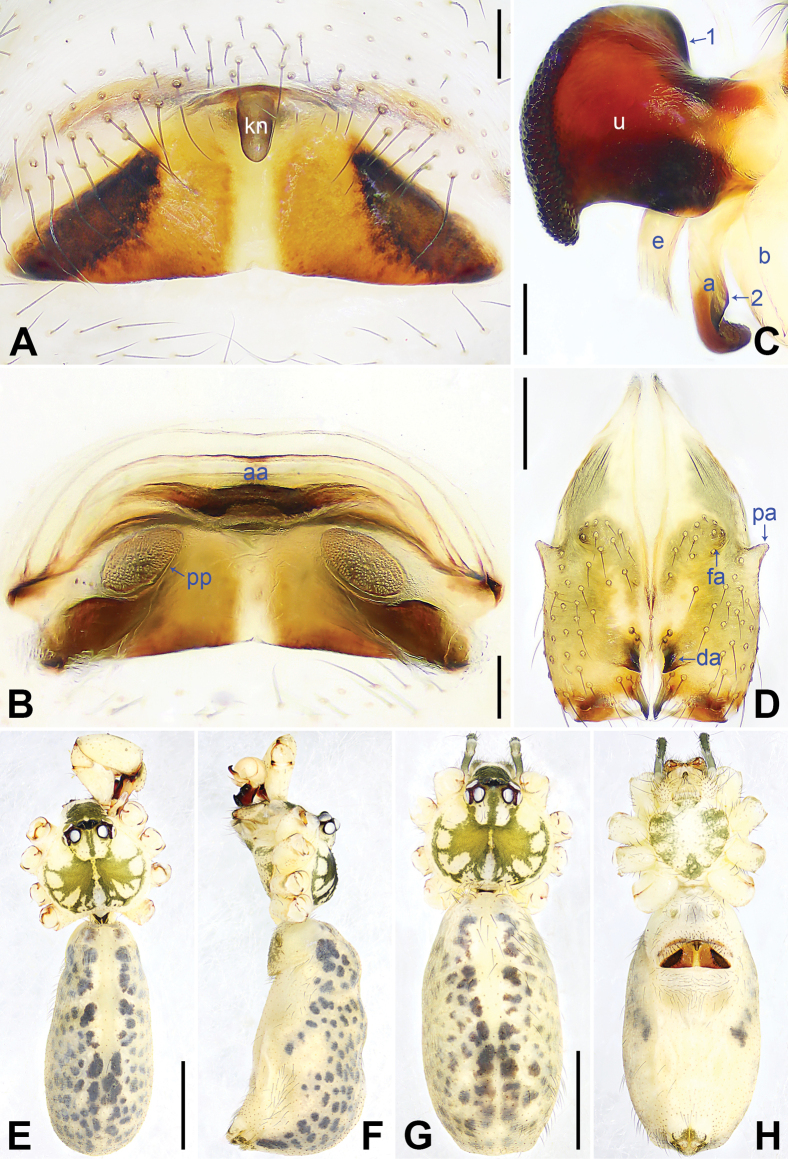
*Pholcusmiyi* sp. nov., holotype male (**C–F**) and paratype female (**A, B, G, H**) **A** epigyne, ventral view **B** vulva, dorsal view **C** bulbal apophyses, prolateral view, arrow 1 points at proximal apophysis, arrow 2 points at angular median branch **D** chelicerae, frontal view **E–H** habitus (**E, G** dorsal view **F** lateral view **H** ventral view). Abbreviations: a = appendix, aa = anterior arch, b = bulb, da = distal apophysis, e = embolus, fa = frontal apophysis, kn = knob, pa = proximo-lateral apophysis, pp = pore plate, u = uncus. Scale bars: 0.10 (**A–C**); 0.20 (**D**); 1.00 (**E–H**).

The new species resembles *P.luding* Tong & Li, 2010 (Tong and Li 2010: 47, figs 2J–L, Q, 11A–F) by having similar uncus (Figs 11C, 14E), male chelicerae (Fig. 11D) and epigyne (Fig. 11A), but can be distinguished by procursus with sclerotized pointed subdistal apophysis (arrow 1 in Fig. 10C vs absent) and sawtooth subdistal membranous process (arrow 2 in Fig. 10C vs absent), by appendix without bifurcated median apophysis (a in Figs 11C, 14E vs present), and by vulval pore plates 3× longer than wide (pp in Fig. 11B vs 2×).

##### Description.

**Male** (***holotype***): Measurements: Total length 3.84 (4.05 with clypeus), carapace 1.06 long, 1.31 wide, opisthosoma 2.78 long, 1.27 wide. Leg I: 36.66 (9.30, 0.58, 9.00, 15.58, 2.20), leg II: 23.31 (6.48, 0.54, 5.58, 9.23, 1.48), leg III: 15.19 (4.55, 0.51, 3.52, 5.51, 1.10), leg IV: 20.00 (5.77, 0.52, 4.80, 7.65, 1.26); tibia I L/d: 75. Eye interdistances and diameters: PME–PME 0.20, PME 0.14, PME–ALE 0.05, AME–AME 0.04, AME 0.10. Sternum width/length: 0.81/0.75.

Color: Carapace yellowish, with brown radiating marks and marginal brown bands; ocular area yellowish, with median and lateral brown bands; clypeus brown; sternum yellowish, with brown marks. Legs yellowish, but dark brown on patellae and whitish on distal parts of femora and tibiae, with darker rings on proximal, subproximal, submedian and subdistal parts of femora and proximal and subdistal parts of tibiae. Opisthosoma yellowish, with dorsal and lateral brown spots.

Body: Habitus as in Fig. 11E, F; ocular area elevated, without eye-stalks.

Chelicerae: As in Fig. 11D, with pair of proximo-lateral apophyses, pair of distal apophyses with two teeth each, and pair of frontal apophyses.

Palp: As in Fig. 10A, B; trochanter with long (4× longer than wide), retrolaterally strongly bulged ventral apophysis; femur with retrolatero-proximal protrusion (arrow in Fig. 10B) and distinct ventral protrusion; tibia with prolatero-ventral protrusion (arrow in Fig. 10A); procursus simple proximally but complex distally, with raised, prolatero-subdistal membranous edge bearing sclerotized pointed subdistal apophysis, sawtooth subdistal membranous process and distal membranous process (arrows 1–3 in Fig. 10C), sawtooth distal apophysis (arrow 4 in Fig. 10C), dorso-subdistal sclerite (arrow 5 in Fig. 10C), and one slender and two strong dorsal spines (arrows in Fig. 10D); uncus with scaly edge and proximal apophysis (arrow 1 in Fig. 11C; arrow in Fig. 14E); appendix hooked, with angular median branch (arrow 2 in Fig. 11C); embolus weakly sclerotized, with some transparent distal projections (Figs 11C, 14E).

Legs: Retrolateral trichobothrium on tibia I at 6% proximally; legs with short vertical setae on tibiae, metatarsi, and tarsi; tarsus I with 26 distinct pseudosegments.

**Female** (***paratype***, SYNU-Ar00487): Similar to male, habitus as in Fig. 11G, H. Total length 3.82 (3.99 with clypeus), carapace 1.13 long, 1.33 wide, opisthosoma 2.69 long, 1.44 wide; tibia I: 6.92; tibia I L/d: 58. Eye interdistances and diameters: PME–PME 0.19, PME 0.13, PME–ALE 0.04, AME–AME 0.04, AME 0.09. Sternum width/length: 0.82/0.77. Epigyne nearly triangular, laterally strongly sclerotized, with knob (Fig. 11A). Vulva with curved, posteriorly sclerotized anterior arch and pair of nearly elliptic pore plates (Fig. 11B).

##### Variation.

Tibia I in paratype male (SYNU-Ar00486): 8.85. Tibia I in another paratype female (SYNU-Ar00488): 6.41.

##### Habitat.

Underside of overhang on rocky cliffs in the mountain area.

##### Distribution.

China (Sichuan, type locality; Fig. 1).

#### ﻿ Pholcus yaan

Taxon classificationAnimaliaAraneaePholcidae

Li, Li & Yao sp. nov.

C3BF20D9-D118-5DBF-8579-C421A2ECB63E

https://zoobank.org/F034C317-2580-420F-B24E-123C64607BE8

Figs 12 , 13 , 14F

##### Type material.

***Holotype***: China • ♂; Sichuan, Yaan, Lushan County, Longdongpo; 30.089279°N, 102.931493°E; alt. 944 m; 24 May 2024; X. Zhang, Y. Wang & Q. Meng leg.; SYNU-Ar00489. ***Paratypes***: China • 3 ♀; same data as for the holotype; SYNU-Ar00490–92.

##### Etymology.

The specific name refers to the type locality; noun in apposition.

##### Diagnosis.

**Figure 12.**
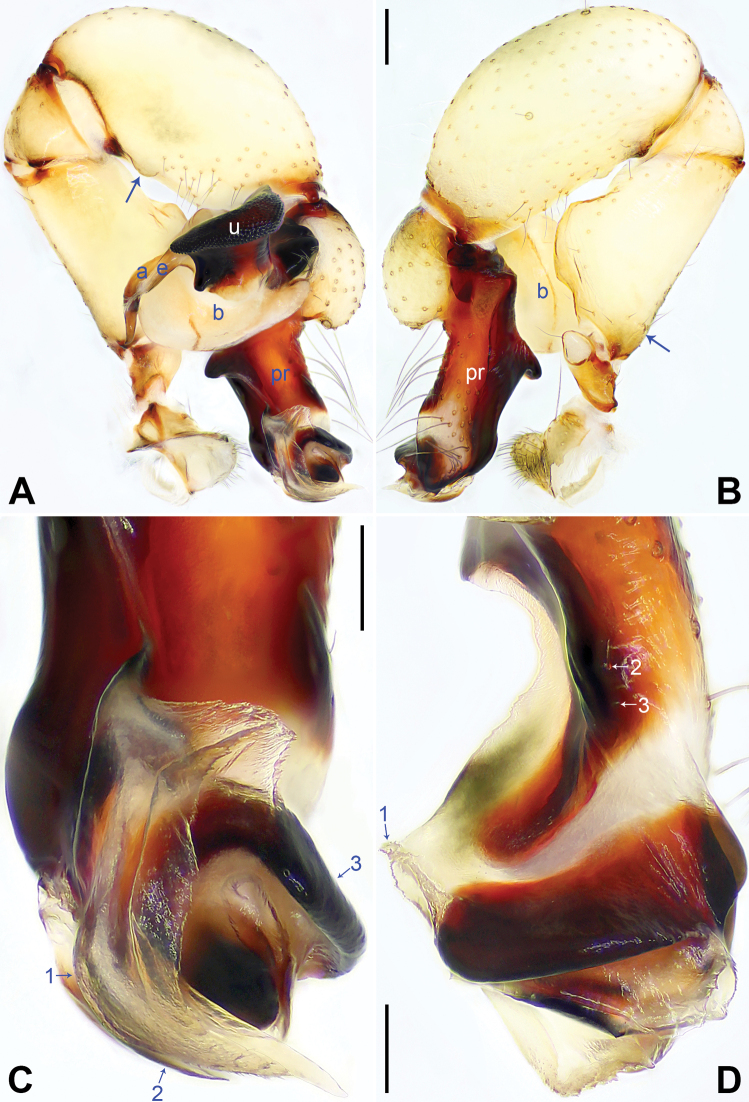
*Pholcusyaan* sp. nov., holotype male **A, B** palp (**A** prolateral view, arrow points at prolatero-ventral protrusion **B** retrolateral view, arrow points at retrolatero-proximal protrusion) **C, D** distal part of procursus (**C** prolateral view, arrow 1 points at distal membranous process, arrow 2 points at sclerotized part, arrow 3 points at sclerotized dorso-subdistal apophysis **D** dorsal view, arrow 1 points at laterally pointed prolatero-subdistal membranous edge, arrows 2, 3 point at dorsal spines). Abbreviations: a = appendix, b = bulb, e = embolus, pr = procursus, u = uncus. Scale bars: 0.20 (**A, B**); 0.10 (**C, D**).

**Figure 13.**
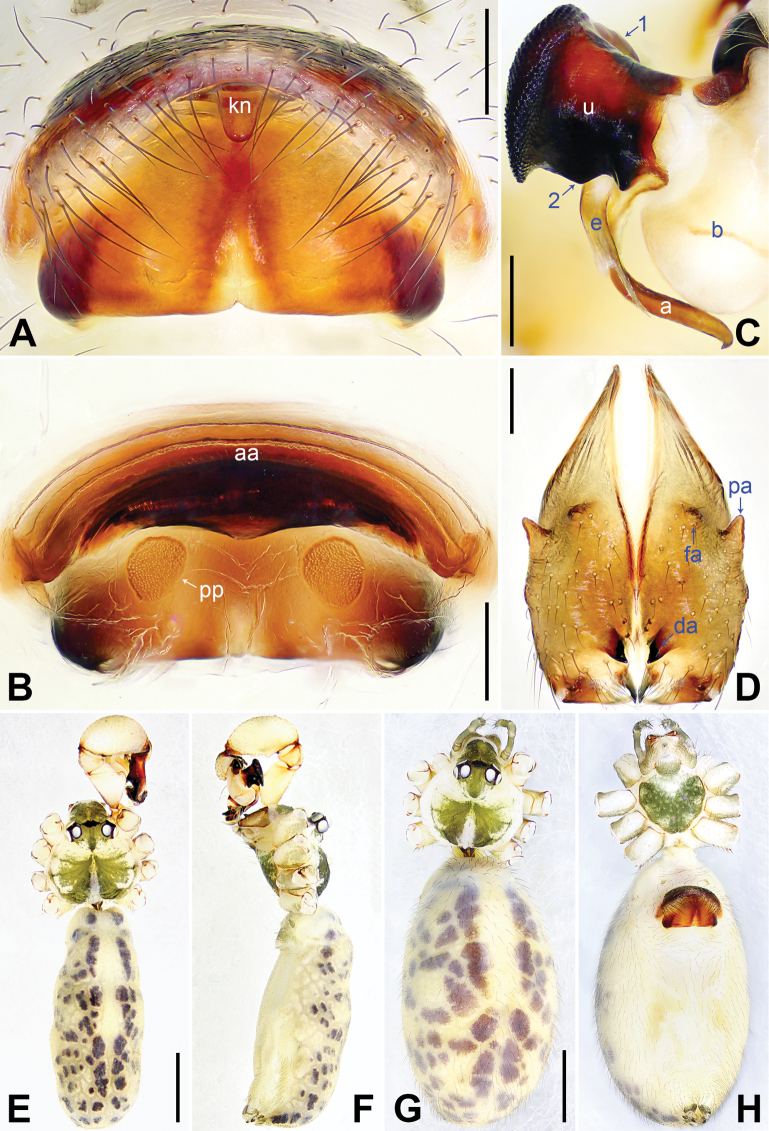
*Pholcusyaan* sp. nov., holotype male (**C–F**) and paratype female (**A, B, G, H**) **A** epigyne, ventral view **B** vulva, dorsal view **C** bulbal apophyses, prolateral view, arrow 1 points at proximal apophysis, arrow 2 points at latero-median protrusion **D** chelicerae, frontal view **E–H** habitus (**E, G** dorsal view **F** lateral view **H** ventral view). Abbreviations: a = appendix, aa = anterior arch, b = bulb, da = distal apophysis, e = embolus, fa = frontal apophysis, kn = knob, pa = proximo-lateral apophysis, pp = pore plate, u = uncus. Scale bars: 0.20 (**A–D**); 1.00 (**E–H**).

**Figure 14.**
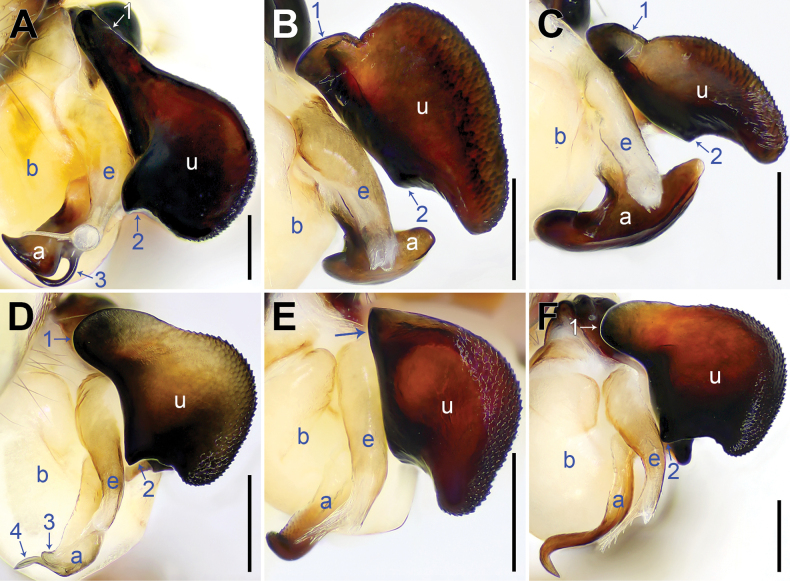
Bulbal apophyses, retrolatero-ventral views **A***Pholcusqiaojia* sp. nov., arrow 1 points at proximal apophysis, arrow 2 points at latero-median protrusion, arrow 3 points at slender subdistal branch **B***P.aba* sp. nov., arrow 1 points at proximal apophysis, arrow 2 points at latero-median protrusion **C***P.wenchuan* sp. nov., arrow 1 points at proximal apophysis, arrow 2 points at latero-median protrusion **D***P.mengding* sp. nov., arrow 1 points at proximal apophysis, arrow 2 points at latero-median protrusion, arrow 3 points at hooked appendix, arrow 4 points at curved subdistal branch **E***P.miyi* sp. nov., arrow points at proximal apophysis **F***P.yaan* sp. nov., arrow 1 points at proximal apophysis, arrow 2 points at latero-median protrusion. Abbreviations: a = appendix, b = bulb, e = embolus, u = uncus. Scale bars: 0.20 (**A–F**).

The new species resembles *P.mengding* sp. nov. (Figs 8A–D, 9A–H, 14D) by having similar uncus (Figs 13C, 14F), male chelicerae (Fig. 13D) and epigyne (Fig. 13A), but can be distinguished by prolatero-subdistal membranous edge of procursus laterally pointed (arrow 1 in Fig. 12D vs laterally blunt), by appendix without branch (a in Figs 13C, 14F vs with curved subdistal branch), and by vulval pore plates nearly round (pp in Fig. 13B vs nearly elliptic, 2× longer than wide).

##### Description.

**Male** (***holotype***): Measurements: Total length 4.64 (4.86 with clypeus), carapace 1.34 long, 1.48 wide, opisthosoma 3.30 long, 1.52 wide. Leg I: 43.27 (10.71, 0.68, 10.77, 18.65, 2.46), leg II: 28.33 (7.69, 0.67, 7.05, 11.54, 1.38), leg III: 19.25 (5.64, 0.56, 4.68, 7.37, 1.00), leg IV: 25.63 (7.37, 0.63, 6.35, 9.94, 1.34); tibia I L/d: 80. Eye interdistances and diameters: PME–PME 0.31, PME 0.18, PME–ALE 0.06, AME–AME 0.07, AME 0.09. Sternum width/length: 1.00/0.90.

Color: Carapace yellowish, with brown radiating marks and marginal brown bands; ocular area and clypeus brown; sternum yellowish, with brown marks. Legs yellowish, but dark brown on patellae and whitish on distal parts of femora and tibiae, with darker rings on subdistal parts of femora and proximal and subdistal parts of tibiae. Opisthosoma yellowish, with dorsal and lateral brown spots.

Body: Habitus as in Fig. 13E, F; ocular area elevated, each eye triad on top of laterally directed eye-stalk.

Chelicerae: As in Fig. 13D, with pair of proximo-lateral apophyses, pair of distal apophyses with two teeth each, and pair of frontal apophyses.

Palp: As in Fig. 12A, B; trochanter with long (2× longer than wide), retrolaterally strongly bulged ventral apophysis; femur with retrolatero-proximal protrusion (arrow in Fig. 12B) and distinct ventral protrusion; tibia with prolatero-ventral protrusion (arrow in Fig. 12A); procursus simple proximally but complex distally, with raised, prolatero-subdistal membranous edge bearing distal membranous process (arrow 1 in Fig. 12C), distal membranous process bearing sclerotized part (arrow 2 in Fig. 12C), sclerotized dorso-subdistal apophysis (arrow 3 in Fig. 12C), and two slender dorsal spines (arrows 2, 3 in Fig. 12D); uncus with scaly edge, proximal apophysis, and distinct latero-median protrusion (arrows 1, 2 in Figs 13C, 14F); appendix slender and curved (Figs 13C, 14F); embolus weakly sclerotized, with some transparent distal projections (Figs 13C, 14F).

Legs: Retrolateral trichobothrium on tibia I at 6% proximally; legs with short vertical setae on tibiae, metatarsi, and tarsi; tarsus I with 37 distinct pseudosegments.

**Female** (***paratype***, SYNU-Ar00490): Similar to male, habitus as in Fig. 13G, H. Total length 5.10 (5.35 with clypeus), carapace 1.22 long, 1.45 wide, opisthosoma 3.88 long, 2.28 wide; tibia I: 8.40; tibia I L/d: 60. Eye interdistances and diameters: PME–PME 0.19, PME 0.17, PME–ALE 0.04, AME–AME 0.06, AME 0.08. Sternum width/length: 0.95/0.89. Ocular area without eye-stalks. Epigyne nearly triangular, laterally strongly sclerotized, with knob (Fig. 13A). Vulva with curved, posteriorly sclerotized anterior arch and pair of nearly round pore plates (Fig. 13B).

##### Variation.

Tibia I in the other two paratype females (SYNU-Ar00491–92): 8.72, 8.91.

##### Habitat.

Underside of overhang on rocky cliffs in the mountain area.

##### Distribution.

China (Sichuan, type locality; Fig. 1).

## ﻿Discussion

*Pholcus* exhibits a high degree of diversity in eastern Sichuan, and our study has identified five newly described species and one newly recorded species from this region. As of now, a total of 19 species have been recorded in eastern Sichuan. However, the survey efforts for *Pholcus* in this area have been highly uneven, primarily because the majority of these species are distributed in the western part of the Sichuan Basin. The current distribution pattern of this genus in eastern Sichuan indicates that it is likely also present in the highlands of the southwestern and northeastern parts of the Sichuan Basin. We are confident that a significant number of new *Pholcus* species remains to be discovered in these regions.

## Supplementary Material

XML Treatment for Pholcus

XML Treatment for Pholcus qiaojia

XML Treatment for Pholcus aba

XML Treatment for Pholcus wenchuan

XML Treatment for Pholcus mengding

XML Treatment for Pholcus miyi

XML Treatment for Pholcus yaan

## ﻿Additional information

### Author ORCIDs

Jinglin Li https://orcid.org/0009-0008-3932-309X

Shuqiang Li https://orcid.org/0000-0002-3290-5416

Xiaoqing Zhang https://orcid.org/0000-0003-0687-9266

Zhiyuan Yao https://orcid.org/0000-0002-1631-0949

### Data availability

All of the data that support the findings of this study are available in the main text.
